# 微孔有机网络材料的合成方法及其在样品前处理中的应用进展

**DOI:** 10.3724/SP.J.1123.2023.07003

**Published:** 2023-12-08

**Authors:** Tao YU, Li CHEN, Wenmin ZHANG, Lan ZHANG, Qiaomei LU

**Affiliations:** 1.福州大学生物科学与工程学院,福建福州350116; 1. College of Biological Science and Engineering, Fuzhou University, Fuzhou 350116, China; 2.福州大学福建省高校测试中心,福建福州350116; 2. Fujian College Association Instrumental Analysis justify of Fuzhou University, Fuzhou 350116, China; 3.食品安全与生物分析教育部重点实验室,福州大学化学学院,福建福州350116; 3. Key Laboratory for Analytical Science of Food Safety and Biology (Ministry of Education), College of Chemistry, Fuzhou University, Fuzhou 350116, China; 4.闽江师范高等专科学校化学与生物工程系,福建福州350108; 4. Division of Chemical and Biological Engineering, Minjiang Teachers College, Fuzhou 350108, China

**Keywords:** 微孔有机网络, Sonogashira反应, 样品前处理, 固相萃取, 吸附剂, 综述, microporous organic networks (MONs), Sonogashira reaction, sample pretreatment, solid phase extraction, adsorbent, review

## Abstract

样品前处理是色谱分析中必不可少的环节。固相萃取是一类应用广泛的前处理方法,吸附剂的优劣直接影响萃取过程对目标化合物的吸附和富集效率,并影响前处理及后续分析方法的灵敏度和选择性。因此吸附剂的选择和开发成为一个研究热点。微孔有机网络(microporous organic networks, MONs)是由芳香炔烃和芳香卤化物通过Sonogashira反应合成的一类新型共价有机材料,具有结构可修饰、比表面积大、孔隙率高、合成简单等优点。本文概述了MONs的合成和功能化修饰方法,着重介绍了该材料在样品前处理领域的应用新进展,并对其发展趋势进行了展望。在合成方法方面,MONs材料的制备从回流合成法、溶剂热合成法发展到室温合成法,合成条件趋向于更温和、更高效。在材料功能化修饰方面,引入大分子物质以及氨基、羟基、羧基等活性基团,能增加MONs材料的选择性和作用位点;将MONs与Fe_3_O_4_、SiO_2_、MOFs结合,形成核壳结构MONs,在此基础上进行煅烧和刻蚀,可形成多孔碳结构或空心多层材料。上述功能化修饰的MONs及其复合材料和目标物之间存在多重作用机制(氢键、疏水、静电、*π-π*相互作用等),因此能实现各类化合物的高效萃取。将MONs作为吸附剂材料应用于固相萃取、固相微萃取、分散固相萃取、磁性固相萃取等多种前处理方法,结合色谱、色谱-质谱联用等技术,获得了较好的吸附效果和较高的灵敏度,展现了MONs材料在样品前处理领域的应用潜力。

## Abstract (en)

Sample pretreatment is an essential step in chromatographic analysis. Solid phase extraction (SPE) is a widely used sample pretreatment method. In SPE, the quality of the adsorbent directly affects the adsorption and enrichment efficiency of the target compounds as well as the sensitivity and selectivity of the pretreatment and subsequent analysis. Therefore, the selection and development of adsorbents has become a research hotspot. Microporous organic networks (MONs) are a novel type of covalent organic materials that are synthesized by the Sonogashira reaction of aromatic alkynes and aromatic halides. These networks have the advantages of modifiable structures, large specific surface areas, high porosity, and simple synthesis methods. This paper reviews the synthesis and functional modification methods of MONs, with an emphasis on their applications in sample pretreatment. Future development trends are also prospected. In terms of synthesis, the preparation methods for MON-based materials have progressed from reflux and solvothermal synthesis to room-temperature synthesis, the conditions of which tend to be milder and more efficient. In terms of functional modification, the introduction of macromolecules and active groups (including amino, hydroxyl, and carboxylic groups) can increase the selectivity and active sites of MON-based materials. The combination of MONs with Fe_3_O_4_, SiO_2_, and metal organic frameworks yields core-shell-structured MONs. Furtherly, they can be calcined and etched to form porous carbon structures or hollow multilayer materials. Functionalized MONs and their composite materials have multiple interaction mechanisms (e. g., hydrogen bonding, hydrophobic, electrostatic, and *π-π* interactions) with various target compounds, thereby realizing their efficient extraction. MONs can be used as adsorbent materials in SPE, Solid phase microextraction, dispersed solid phase extraction, magnetic solid phase extraction, and other pretreatment methods. When combined with chromatography and chromatography-mass spectrometry, MONs achieve good adsorption effects and high sensitivity, demonstrating the application potential of these materials in sample pretreatment.

2007年,Cooper等^[1]^首次利用多乙炔基取代芳基化合物和多碘(溴)取代芳基化合物,通过Sonogashira反应合成了一系列共轭微孔聚合物(conjugated microporous polymer, CMP)材料。该类CMP材料就是微孔有机网络(microporous organic networks, MONs)的最初雏形,而MONs属于CMP材料的一个子类。2012年,Son团队^[2]^首次正式命名MONs材料。它是以芳香炔烃作为单体、卤化芳基作为连接剂在碱性条件下通过Sonogashira反应合成的一类多孔有机材料^[3]^。

和当下研究相当活跃的金属有机骨架(metal organic framework, MOFs)、共价有机骨架(covalent organic framework, COFs)类似,MONs材料也具有比表面积大、结构可调控、稳定性良好等突出优势,同时克服了MOFs材料对环境(水分、氧气等)敏感、COFs材料质量较轻不易离心等不足。该类材料的出现,也改变了之前学术界普遍认为只有长程有序的结晶态聚合物(包括MOFs、COFs)才能实现可控尺寸的观点。近年来,MONs已在催化^[4,5]^、传感^[6,7]^、锂离子电池^[8,9]^、药物递送^[10,11]^、发光^[12,13]^、气体储存^[14,15]^等领域得到广泛应用。文献调研发现,国外Cooper和Son团队在MONs合成和应用上做了诸多开创性工作,而国内以严秀平、杨成雄和赵汝松等课题组为主,扩大了MONs在分析化学方面的应用。

## 1 MONs合成原理及方法

### 1.1 合成原理

Sonogashira反应^[16]^是含炔单体和卤代芳烃在钯/铜盐催化剂作用下的一类交叉偶联反应。MONs正是基于Sonogashira反应设计合成的一类新兴多孔材料(见图1)。常见的芳香炔基单元有线性、三角形、四边形及四面体结构,而芳基卤化物常见以碘代、溴代为主。Cooper等研究者发现,基于碘化单体的合成材料反应速率、缩合程度和比表面积略高于溴代单体^[17⇓⇓-20]^。然而,商品化的二碘类单体较少,因此以溴代芳烃作为单体的研究居多。Cui等^[21]^详细讨论了MON的拓扑学结构,且采用不同的构筑单体,构建了结构多样的MONs^[22⇓⇓⇓-26]^。此外,MONs可以与不同的物质交联,形成MONs复合材料^[27⇓⇓-30]^,极大地丰富了MONs的种类和性质。

**图1:**
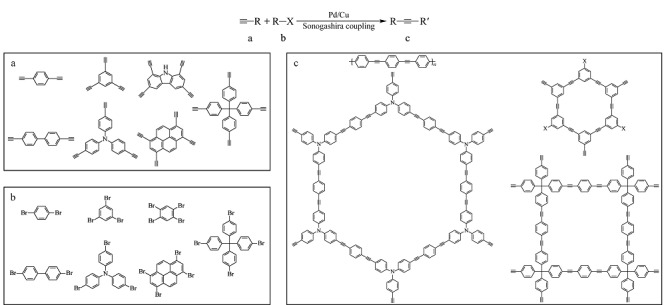
基于Sonogashira反应合成MONs

### 1.2 合成方法

#### 1.2.1 回流合成法

早期主要基于回流法合成MONs材料。2007年,Cooper等^[1]^首次合成几种不同类型的CMP时,将1,3,5-三乙炔苯、1,4-二碘苯、四(三苯基膦)钯、碘化铜溶解在甲醇和三乙胺的混合溶液中,加热至80 ℃,在氮气气氛下搅拌72 h冷却到室温后,相继用氯仿、甲醇和丙酮等溶剂洗涤以去除残留物,通过甲醇索氏萃取48 h对聚合物进一步纯化。最后得到微孔结构的棕色聚合物,比表面积高达834 m^2^/g。之后的两三年,合成MONs材料大多基于80~120 ℃条件下回流24~72 h,该制备方法耗时长、有机溶剂消耗大。

#### 1.2.2 无铜溶剂热合成法

Trunk等^[31]^对Sonogashira反应法进行改进,先将1,3,5-三乙炔苯和1,3,5-三碘苯等物质的量加入,再加入四(三苯基膦)钯、二甲基甲酰胺和三乙胺,100 ℃油浴加热,获得高比表面积的MONs。该反应既避免了使用碘化铜,又减少了钯催化剂的用量(物质的量占比低至0.65%),合成过程用料减少、更为简单。但后续研究发现,大多数无铜反应仍需要加入其他活化剂(包括卤化锌、氧化银等)^[32]^作为替代,限制了无铜反应的进一步推广。

#### 1.2.3 室温合成法

近十年来,室温合成法获得快速发展。杨成雄团队^[33,34]^将三乙胺和甲苯预混合,再加入含炔单体、卤化芳基以及碘化铜、双(三苯基膦)二氯钯两种催化剂,将混合物在室温下搅拌数小时,最后离心、洗涤和干燥。该课题组^[35]^还重点比较了合成方法对MONs复合物材料形貌和萃取效率的影响。结果发现,室温合成法与传统回流法效果相当,但室温法更加简单、温和、高效,已成为合成MONs最常用的方法。

## 2 MONs材料的功能化修饰方法

2019年,Cui等^[34]^以四烷基(4-乙基苯基)甲烷和1,4-二碘苯为原料,在室温下合成了纯MON用于吸附水溶液中的四溴双酚A(TBBPA),吸附容量高达227.3 mg/g,吸附机理主要涉及*π-π*堆叠和疏水作用。然而,纯MONs与目标物的相互作用力有限,且材料密度较低,从样品基质中不易收集和回收。因此,很有必要对MONs进行后修饰以提高对目标物的提取效率以及材料在样品基质中的分离能力。

### 2.1 引入活性基团

MONs具有刚性平面和共轭体系,通过在反应单体中引入一种或多种羧基、羟基、氨基、磺酸基等活性基团对MONs进行修饰,可以增强其亲水性,增加吸附作用位点。

Han等^[36]^采用室温搅拌法分别合成了MON、MON-COOH、MON-2COOH和MON-4COOH几种吸附材料。结果发现,羧基的引入有效增加了材料和目标物的相互作用位点。同时,羧基数量越多,富集效果越好。之后,将MON-2COOH^[37]^和MON-4COOH^[38]^等材料进一步应用于水中苯并三唑类污染物和阳离子染料的快速吸附和去除,最大吸附量分别为369 mg/g和3126 mg/g。羧基的引入扩大了功能化MONs材料在环境污染物分析领域的应用。

2022年,Liu等^[39]^选择2,4,6-三溴苯酚和1,3,5-三(4-乙炔基苯基)苯两种单体,成功制备了MON-OH并用于三嗪类除草剂的吸附萃取。5种三嗪类目标物均含有5个氢键受体和2个氢键供体,分子半径为0.39~0.42 nm,容易被MON-OH选择性吸附(材料孔径约1.86 nm),方法回收率高达99.8%~114.7%。吸附机理显示,MON-OH与含卤目标物之间不仅形成-OH…N-和-NH…O-等氢键,还可形成-O…Cl-键。

同年,Li等^[24]^选择2,5-二溴苯胺和四(4-乙炔基苯基)甲烷合成MON-NH_2_并用作固相微萃取(SPME)的吸附剂。MON-NH_2_对8种酚类内分泌干扰物(EDCs)的吸附量在初始阶段快速增加,吸附等温线符合Langmuir模型,吸附动力学遵循伪二阶模型。该方法定量限为0.005 μg/L,回收率为87.0%~112.5%,进一步验证了氨基基团的引入,能有效增加材料对酚类目标物的氢键吸附位点,从而提高萃取选择性。

鉴于MONs材料中含有丰富的炔烃基团,He等^[26]^将巯基-炔“点击”化学后修饰策略应用于样品前处理领域,在磁性MON(MMON)的基础上创新性制备了含有磺酸基的MMON-SO_3_H@SO_3_Na磁性复合材料。该材料的吸附效果和富集倍数均优于其他3种吸附材料(Fe_3_O_4_、MMON、MMON-SO_3_H等),表明MON的共轭芳香骨架和磺酸盐基团在萃取过程中起到重要作用,实现了对复杂果蔬样品中痕量苯并咪唑杀菌剂的高效吸附和检测。该研究基于“点击”化学反应,进一步拓宽了MONs功能化修饰的方法。

在单官能团化基础上,掺杂多种官能团可进一步增加吸附位点,协同提高吸附效果。2021年,Han等^[40]^合成了同时富含氨基和羟基的双功能化MON(B-MON),丰富的多孔结构以及多重吸附机制使其特别适合吸附含有苯环、萘环、羟基、羧基等基团的分析物。在此基础上,Yu等^[33]^基于室温合成法制备了类似双官能团复合物(MMON-B),合成过程更加简单温和、绿色环保。球形MON-B孔径约1.2 nm,比表面积约140.4 m^2^/g。双官能团的引入能增加吸附过程中的作用位点,吸附效果优于单官能团吸附剂(MMON-OH和MMON-NH_2_)。

### 2.2 引入大分子物质

环糊精(CD)是一类环状低聚糖,特有的疏水内腔和亲水外腔使其拥有独特的主客体识别能力,有助于增强方法的选择性。将CD和MON材料相结合,能有效弥补MON对亲水物质结合力弱的缺陷。2022年,Cui等^[29]^基于一种溶剂调节的无模板策略合成了新的二维环糊精MON纳米片(2D CD-MON)。对比了不同反应溶剂(四氢呋喃(THF)、*N*,*N*-二甲基甲酰胺(DMF)、甲苯(PhMe))对材料合成过程的影响,发现溶剂组成对CD-MON的厚度、聚集性以及孔洞结构都有重要的调控作用。经过实验优化,发现CD-MON-DMF具有最高的比表面积、孔隙体积和孔径分布。以双酚AF (BPAF)作为模型污染物制备的CD-MON-DMF对BPAF具有超快的吸附动力学(10 s)和极大的饱和吸附量(327.9 mg/g)。该研究为二维MONs的选择性设计开辟了新思路,也揭示了形貌控制在吸附应用中的重要性。同年,Li等^[28]^自制了薄片状CD-MON材料,比表面积为141.8 m^2^/g,对双酚A(BPA)的吸附效率明显优于其他环糊精类吸附剂。此外,该材料具有良好的再生性能,是一种很有前途的废水修复材料。

2023年,Wang等^[41]^利用18-冠醚-6与芳香炔基之间的偶联合成了3种拓扑结构的冠醚基MON。冠醚(CE)拥有环状空腔,可用于捕获金属离子或有机分子,将CE与疏水MONs结合是提高CE稳定性的可行方法。研究表明,表面吸附和颗粒内扩散共同发挥重要作用,冠醚基MON对2,4,6-三氯苯酚(2,4,6-TCP)的最大吸附容量达到187.9 mg/g。这是冠醚类MONs的首例研究,拓宽了MONs的选择性应用。

此外,赵汝松课题组^[30,42]^合成了基于聚苯的共轭微孔聚合物(PP-CMPs)。该材料在三维聚苯支架上进行*π-π*共轭,具有固有微孔结构和较大表面积^[43]^,实现了水样中对苯氧基羧酸除草剂、酚类物质的高灵敏度分析。

### 2.3 核壳结构MONs

基于模板合成法,以常见的Fe_3_O_4_、SiO_2_、MOFs为基体和内核,MONs作为外壳,形成核壳结构的MONs复合材料,是目前MONs功能化修饰最为丰富的一种形式。通过改变核或壳的数量和种类,利用不同材料的各自优势,理论上可能获得更优异的吸附性能。

#### 2.3.1 以Fe_3_O_4_为核

作为目前应用最多的磁性吸附剂基体,裸露的Fe_3_O_4_纳米颗粒表面活性基团较少,在水溶液中易产生聚集或氧化现象,影响其稳定性和萃取效果。因此,研究者们以Fe_3_O_4_为磁芯,通过单体引入法和外源引入法,制备了一系列磁性MONs复合物。

杨成雄课题组^[44,45]^合成了Fe_3_O_4_@MON-NH_2_,将其作为磁性吸附剂分别用于吸附水样中EDCs和TBBPA。所建立的方法具有检出限低、富集系数大、前处理过程简单等优点。通过疏水作用和氢键作用,Fe_3_O_4_@MON-NH_2_吸附TBBPA在1 min内达到平衡,最大吸附量为135.9 mg/g。随后,课题组继续尝试在Fe_3_O_4_磁球上包覆其他材质。聚多巴胺(PDA)具有独特的生物相容性和黏附性,可作为氧化铁基质与萃取介质间良好的结合基质。PDA在Fe_3_O_4_上自聚合,可产生均匀的PDA涂层用于支持疏水萘环MON(NMON)壳的生长。最终制备的磁性NMON复合材料能高效吸附痕量羟基多环芳烃和对硝基苯酚,富集倍数为92.6~98.4,检出限为0.055 μg/L^[46]^。与PDA相类似,聚乙烯亚胺(PEI)含有丰富的氨基和乙烯基,能提供更多氢键和疏水作用位点。Fe_3_O_4_@MON带负电荷,与带正电荷的PEI之间存在静电引力,因此可以通过一锅煮法将PEI直接包覆到Fe_3_O_4_@MON表面,合成Fe_3_O_4_@MON-PEI_600_^[27]^用于吸附非甾体抗炎药(NSAIDs)。该材料对目标物的萃取效率远高于Fe_3_O_4_@PEI_600_和Fe_3_O_4_@MON,这主要得益于MON的共轭疏水结构和PEI_600_的丰富氨基。

#### 2.3.2 以多孔纳米硅球(SiO_2_)为核

SiO_2_比表面积大、表面易修饰,是制备各种复合材料的常用基体之一。用MON改性SiO_2_微球,得到均匀且分散、背压小的MON@SiO_2_,是在线固相萃取和高效液相色谱(HPLC)固定相的良好材料。

以固定相材料为例,Du等^[47]^和Yu等^[48]^基于MON@SiO_2_材料,自制HPLC色谱柱,分离几类小分子烷烃、酚类和芳烃化合物,获得了较高的分离效率。Yang等^[49]^进一步探索MON@SiO_2_固定相对分析物的分离适用性。分离对象不仅包括7大类小分子化合物(多环芳烃(PAHs)、NSAIDs、EDCs等),还包括蛋白质大分子。结果发现,该新型MON@SiO_2_材料对多类目标物的分离性能优于商用C_18_色谱柱,最佳柱效高达17880塔板数/米(正丁苯)。该研究揭示了MONs材料在大分子分离分析方面的巨大潜力。为提高固定相亲水性,该课题组继续引入羧基和巯基琥珀酸(MER),制备了核-壳结构MON-2COOH@SiO_2_^[50]^和MON-2COOH@SiO_2_-MER^[51]^,应用于混合模式色谱时,对各种疏水和亲水分析物都具有良好的分离性能。

#### 2.3.3 以MOFs为核

众所周知,MOFs材料结构丰富多样、比表面积大、易于功能化修饰,是一类优异的吸附材料。然而,MOFs在潮湿环境下存在竞争性吸湿和框架结构坍塌等问题,若在MOFs表面生长一层MON材料,形成疏水“屏障”,就可有效提高MOFs的水稳定性。鉴于此,将二者杂化制备的MOFs@MON复合材料往往能提高吸附效果。

2014年,Son团队^[52]^以四(4-乙炔基苯基)甲烷、2,5-二碘苯、UiO-66-NH_2_等原料为反应体系,通过优化不同物料之间的配比,合成了多种MOF@MON材料(MOF@MON-1、MOF@MON-2、MOF@MON-3和MOF@MON-4,比表面积为703~895 m^2^/g, MON层厚度约为8~30 nm)。该类UiO-66-NH_2_@MON复合材料具有疏水表面和微孔结构,能实现对甲苯的大容量吸附。该研究开启了MONs材料在分析化学领域应用的先河。

Jia等^[53]^将MOF@MON复合物固定在不锈钢纤维上,分别制备了MOF-5@MON、MIL-101@MON用于萃取和富集水、环境及食品中的PAHs,富集因子高达1215~3805。Li等^[54]^采用两步原位生长法,在Fe_3_O_4_@SiO_2_上先包覆一层UiO-66-NH_2_,再以Fe_3_O_4_和MOFs为核,继续生长MON形成磁性复合多孔框架材料(Fe_3_O_4_@UiO-66-NH_2_@MON)。MON层的引入使复合材料的比表面积从254.3 m^2^/g增加到418.4 m^2^/g,从而增强了该复合材料对黄曲霉毒素的吸附效果。结合液相色谱-荧光检测法,检出限为0.15~0.87 μg/L,并成功地用于谷物中生物毒素残留的高灵敏度检测。

### 2.4 MONs的煅烧和刻蚀

在上述核壳结构基础上,将MONs复合材料进一步煅烧碳化和刻蚀,通过改变它们的形貌特征及结构性质,以此产生不同的吸附效果。

MONs材料含碳量较高,是理想的碳化前驱体。2014年,Son课题组^[8]^率先在Fe_3_O_4_表面生长MON,再将此复合材料置于氩气下600~800 ℃煅烧碳化,得到的多孔碳(Fe_3_O_4_@C)可作为电荷存储材料。研究表明,该材料在700 ℃的条件下煅烧得到的Fe_3_O_4_@C-700有最出色的性能,库仑效率保持在97%以上,其性能优于或相当于以葡萄糖或聚合物为碳源制备的Fe_3_O_4_-碳材料。He等^[55]^在MON-2COOH上修饰Fe^3+^并煅烧制备出磁性多孔碳(MPC)衍生物,对阳离子染料具有强吸附能力。将MON-OH在500 ℃下进行煅烧^[56]^制备的MON-OH-C材料的比表面积、孔体积和吸附量均显著高于MON-OH材料,证实MONs碳化对增大比表面积和提高吸附性能确实有重要作用。

在核壳结构的基础上把SiO_2_、MOFs等模板刻蚀,得到中空结构的MON(H-MON)。相比于实心结构,中空结构比表面积利用率更高、传质距离更短。2013年,Son课题组^[4]^率先提出一种形状控制的合成方法。基于室温搅拌法在SiO_2_上生长MON,再用氢氟酸(HF)刻蚀硅球得到H-MON。在有机金属前驱体(八羰基钴)的作用下将钴引入到H-MON中,由于模板的孔隙性,可以最大限度地扩大材料的表面积,而有机模板可以通过空气热处理去除,从而获得形状可控的纳米空心Co_3_O_4_,在H_2_O_2_氧化中表现出优异的催化活性。Li等^[57]^采用类似方法制备了H-MON,用作SPME吸附剂提取短链氯化石蜡。

材料的中空结构能有效提高发光和传感性能。Park等^[6]^通过串联合成法制备了含有异香豆素基团的MON材料(SiO_2_@IC-MON),再用HF刻蚀得到H-IC-MON。异香豆素的引入使得MON的激发波长发生红移,实现了激发光由紫外光向可见光的转变,可以避免在传感过程中有机材料的分解。非空心的IC-MON由于分散能力差无法用于传感,表明H-IC-MON良好的可见光吸收和发射特性是由异香豆素和中空形态共同引起的。

多次重复生长复合和刻蚀的过程,即可制备多层空心材料。Son课题组^[10]^制备了三壳和双壳的中空磺化MON球(TH-SMON和DH-SMON)。具体操作如下:在硅球上生长MON再用氯磺酸刻蚀,再磺化包覆ZIF-8,之后继续生长MON,再刻蚀ZIF-8,重复上述包覆-刻蚀的过程,得到了在水介质中分散良好的多壳中空微孔材料。壳层数越多,载药效率就越高。此外,该课题组^[9]^进一步尝试将刻蚀和煅烧结合起来获得不同性能的MON复合材料。在Cu_2_O模板上生长一层富氮MON,再用盐酸刻蚀内部得到H-NMON,该材料继续在氩气下热处理形成空心掺杂N的碳盒(H-NCBs),它的N掺杂、中空结构和薄壳层可以提供优异的电化学性能,因此可以作为电池中的超级电容器。

## 3 MONs在样品前处理领域中的应用

综上,MON及其复合材料在催化、传感、电池、药物传递等许多领域中已有应用,由于其比表面积大、化学稳定性好、易于功能化修饰,是一类极有潜力的吸附剂,因此也适用于样品前处理领域。固相萃取(SPE)是一类应用广泛的预处理技术,还衍生出SPME、分散固相萃取(dSPE)、磁性固相萃取(MSPE)等多种形式。作为一种新型高效吸附材料,Cui等^[21]^综述了MONs材料在分离分析(色谱分离介质和样品预处理)领域的应用进展。可以说,MONs的筛选和制备仍是当前的研究热点(见图2)。

**图2:**
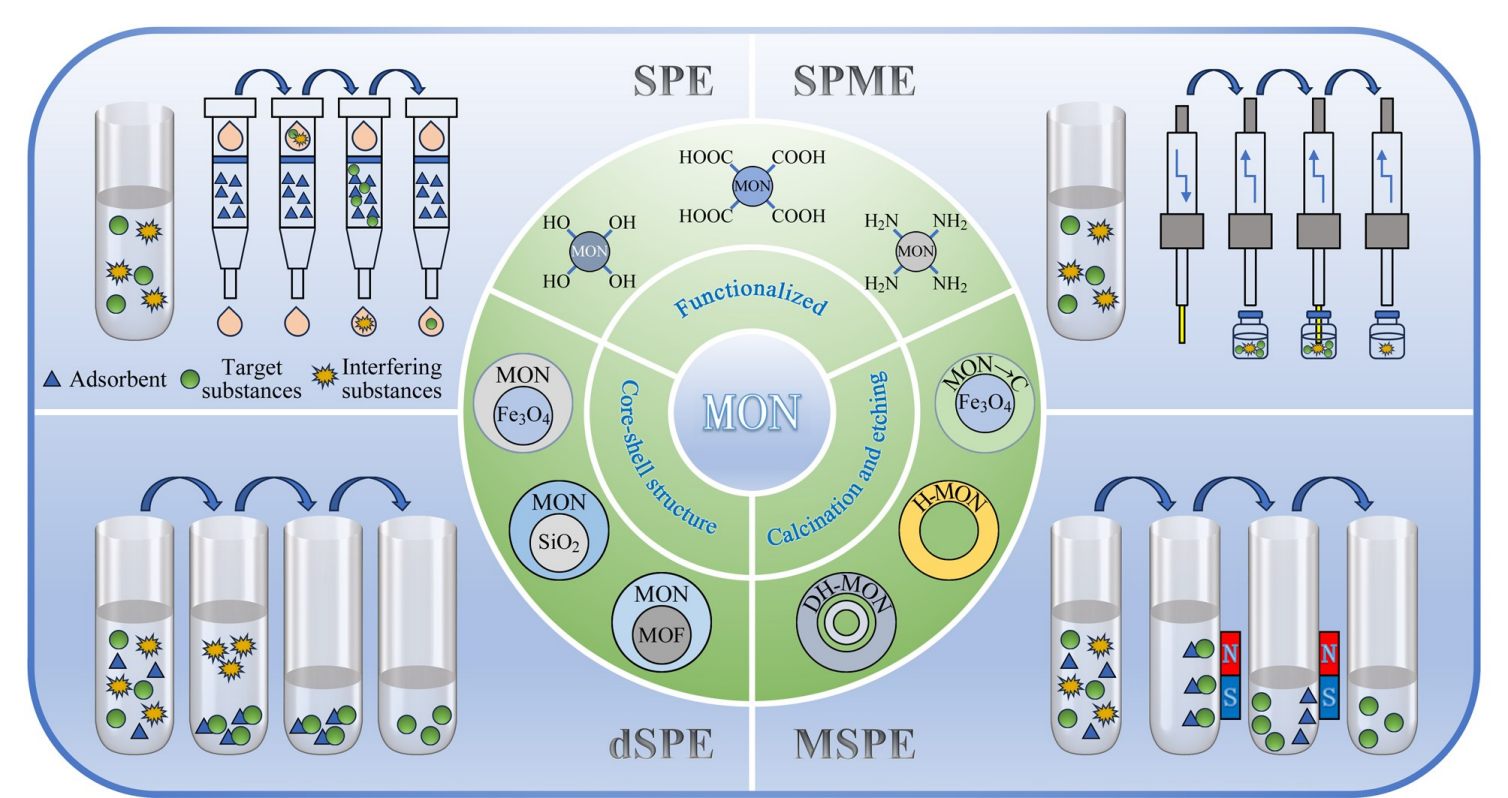
MONs材料在样品前处理中的应用示意图

### 3.1 固相萃取

吸附剂是影响SPE回收率和选择性的关键因素。Li等^[58]^合成了MON-COOH作为SPE小柱填料,选择超纯水活化和淋洗、以甲醇为解吸溶剂结合HPLC测定水样中的苯酚;该方法的线性范围宽、可重复利用性好。Liu等^[39]^合成了MON-OH吸附剂,建立了三嗪类除草剂的MON-OH-SPE-LC-MS/MS检测方法;富集因子高达200,对水样和果汁样品的检出限为0.03~0.15 ng/L和0.04~0.21 ng/L,为食品安全中污染物检测提供了实际解决方法。

在线SPE具有重复性好、易自动化等优势,Du等^[22]^制备了用于在线SPE的SiO_2_@MON-NH_2_微球,与HPLC结合用于分析5种酚类,方法具有很高的富集因子(5036~5689)和较低的检出限(0.009~0.030 μg/L)。

### 3.2 固相微萃取

1990年,Pawliszyn课题组^[59]^首次提出集采样、提取、浓缩、纯化、进样于一体的SPME富集方法。MONs具有良好的热稳定性和化学稳定性,适合作为SPME的吸附剂。不锈钢丝纤维经HF刻蚀,物理粘接或者化学键合上MONs材料,吸附特定目标物后经气相色谱进样口直接解吸、进样分析。陈相峰和赵汝松课题组分别合成了MOF@MON^[53]^、H-MON^[57]^、PP-CMPs^[42]^等材料用作SPME涂层,实现了复杂样品中酚类物质、PAHs、短链氯化石蜡的高效分析。2022年,Li等^[24]^合成了MON-NH_2_材料,结合SPME-GC-MS/MS方法分析EDCs,并建立了上述目标物从安抚奶嘴向唾液模拟物迁移的模型,通过理论计算和实验残留量监测,来预测和评估EDCs的日常暴露风险。在前期基础上,该课题组进一步发展了B-MON包覆、中空纤维膜(HFM)保护的SPME方法^[60]^。B-MON涂层对酚类物质的氢键作用很强,故萃取效果明显优于商用二乙烯基苯-碳-聚二甲基硅氧烷(DVB-CAR-PDMS)涂层。采用中空纤维膜保护后,方法的基质效应明显降低。建立的HFM-SPME-GC-MS/MS法检出限低(0.005 μg/L),基质效应小(母乳:-7.7%~-17.2%,配方奶:-5.0%~-17.9%),使用寿命长(使用次数大于100),并报道了中国婴儿配方奶粉中酚类EDCs的含量情况。

### 3.3 分散固相萃取

分散固相萃取^[61]^是直接将固体吸附剂分散到样品溶液中,在涡旋或超声辅助下吸附目标物,整个过程操作简便、环境友好。

将基于聚苯的共轭微孔聚合物(PP-CMPs)作为dSPE吸附剂^[30]^, PP-CMPs孔径约1.6 nm,大于苯氧基羧酸除草剂分子尺寸(0.80~0.89 nm),使目标物能选择性地进入材料微孔中,结合LC-MS/MS检测,方法检出限为0.55~3.84 ng/L。2022年,Wang等^[62]^借鉴MONs构筑单体中有一类是卤代芳烃的事实,直接以卤化污染物如TBBPA、2,3-二氯酚和2,4,6-三氯酚为起始单体,合成3种相应的MON,反过来又用于这些污染物的快速吸附和去除。该工作特色在于卤化单体和吸附对象是相同物质,污染物洗脱后又可作为合成MON的原料,实现了吸附剂的可回收和再利用,符合绿色化学的需求。然而,MONs及其复合物密度较低,仅靠离心不易使吸附剂与溶液分离,故一定程度上限制了该类材料在dSPE中更广泛的应用。

### 3.4 磁性固相萃取

Fe_3_O_4_在外磁场下能够定向移动且化学性能稳定,常作为MSPE方法中的磁性基底。MONs良好的萃取性能结合磁球良好的分离性能,使磁性MONs材料成为一类高效吸附剂。

2018年,Lei等^[63]^以Fe_3_O_4_为磁芯,在其上生长共轭三维网络制备磁性聚(苯乙炔)MON复合材料,以其作为MPSE的吸附剂富集蔬菜和水果中6种杀菌剂,解吸后进入UPLC-MS/MS测定,方法检出限低,为0.27~3.1 ng/L。2020年,Du等^[44]^以2,5-二溴苯胺取代1,4-二碘苯,将厚度约为40 nm的MON-NH_2_层成功包覆在Fe_3_O_4_磁球上,所制备的材料具有良好的吸附能力,比表面积约370.6 m^2^/g。赵哲^[64]^制备了磁性三嗪基MON复合材料(Fe_3_O_4_@TMON),并对其结构、形貌和性能进行了表征;该材料对3种抗生素的富集因子高达256,并实现了水体和肉类中抗生素残留的分析。

除了以Fe_3_O_4_为磁芯外,近年来也逐渐开发出新的磁芯材料。ZIF-67是由Co(Ⅱ)盐和2-甲基咪唑合成,其炭化导致Co(Ⅱ)聚集为Co磁性纳米颗粒,2-甲基咪唑转化为N掺杂的多孔碳(Co@C或Co@NC)。Son课题组^[65]^将ZIF-67@MON在氩气条件下600 ℃煅烧生成磁性空心Co@C材料,该材料对苯胺和4-硝基苯具有优异的吸附效果和回收率。该项工作是首次热裂解ZIF-MON,设计新型碳-金属复合材料的报道。杨成雄课题组将ZIF-67在氩气下700 ℃高温煅烧,以Co@NC为磁芯原位生长MON-2NH_2_,制备得到Co@NC-MON-2NH_2_。独特的微孔结构和较大的表面积(孔径约1.8 nm,比表面积为316.6 m^2^/g)使其在吸附多类化合物(PAHs、植物生长调节剂、芳香胺等)时表现优异,富集倍数均大于110^[66⇓-68]^。相比Fe_3_O_4_磁芯,Co@NC不仅可以通过煅烧温度来控制Co磁性纳米颗粒产生从而控制磁性的大小,而且煅烧产生的碳纳米管对目标物也有一定的吸附效果。

### 3.5 其他固相萃取方式

各种涂层包裹的内置磁芯的搅拌棒可以在自搅拌过程中萃取分析物。基于此发展起来的搅拌棒吸附萃取(SBSE)技术具有磁分离简单、吸附容量高等优点。杨成雄课题组^[36]^采用室温搅拌法制备了MON-2COOH,并将其用于SBSE富集食品和水样中的苯脲类除草剂,结合HPLC-DAD方法,检出限为0.025~0.070 μg/L。同年,该团队^[40]^进一步合成B-MON材料,其富含氢键位点和多孔结构,对香料和对羟基苯甲酸酯表现出高效的吸附效果,富集倍数大于40倍。

作为SPE技术的微型化发展,管尖固相萃取(PT-SPE)的萃取效率同样取决于涂层的性质。赵哲等^[69]^选择1,3,5-三乙炔苯和2,4,6-三碘苯酚作为单体,室温下快速合成MON-OH作为PT-SPE吸附剂。该材料通过*π-π*作用、氢键作用和疏水作用,能够有效吸附牛奶中的痕量喹诺酮类抗生素,方法回收率优于C_18_、石墨化炭黑、硅酸镁/弗罗里硅土、中性氧化铝等商业化吸附材料。更多基于MONs材料在样品前处理中的应用汇总见表1。

**表1:** MONs材料在样品前处理中的应用

Adsorbents	Pretreatment	Analytical method	Analytes	Samples		EFs		LODs/ (pg/L)		Ref.	
SiO_2_@MON-NH_2_	SPE	HPLC	phenols	water		5036-5689		9-30		[22]	
MON-2OH	dSPE	HPLC	TBBPA	water		-		-		[23]	
MON-NH_2_	SPME	GC-MS/MS	EDCs	pacifiers		-		5		[24]	
TMON	dSPE	UPLC-PDA	FLU, NAD	water		-		-		[25]	
MMONSO_3_H@SO_3_Na	MSPE	HPLC-UV	BZDs	cucumber, tomato, pear		97.8		30-90		[26]	
Fe_3_O_4_@MON-PEI_600_	MSPE	HPLC-UV	NSAIDs	wastewater		97.0-98.2		42-149		[27]	
CD-MON	dSPE	UV	BPA	water		-		-		[28]	
PP-CMPs	dSPE	LC-MS/MS	PCAs	water		-		0.5-3.8		[30]	
MMON-B	MSPE	HPLC-UV	vanillins	milky beverage, milk tea, juice		95.3-98.4		100-150		[33]	
MON	dSPE	HPLC	TBBPA	water		-		-		[34]	
HMON@MIP	dSPE	HPLC-FLD	AFT B1, ST	rice, maize, soybean		-		4.4-6.7		[35]	
MON-2COOH	SBSE	HPLC-PDA	PUHs	apple, tomato, water		46-49		25-70		[36]	
MON-2COOH	dSPE	HPLC	BTri, 5-TTri	water		-		-		[37]	
MON-4COOH	SPE	UV	cationic dyes	water		-		-		[38]	
MON-OH	SPE	LC-MS/MS	triazine herbicides	water, juice		200		0.03-0.21		[39]	
B-MON	SBSE	HPLC-PDA	parabens, flavors	candy rod, lipstick, soybean milk powder		40-49		10-35		[40]	
CE-based MON	dSPE	HPLC	2,4,6-TCP	water		-		-		[41]	
PP-CMPs	SPME	GC-MS/MS	phenols	water		519-2372		0.02-0.05		[42]	
Fe_3_O_4_@MON-NH_2_	MSPE	HPLC-UV	EDCs	water, bottle, juice		172-197		15-30		[44]	
Fe_3_O_4_@MON-NH_2_	MSPE	HPLC-UV	TBBPA	water		-		-		[45]	
Fe_3_O_4_@PDA@NMON	MSPE	HPLC-UV	OH-PAHs, *p*-Npn	wastewater		92.6-98.4		55-90		[46]	
MOF-5@MON, MIL-101@MON	SPME	HPLC-UV	PAHs	water, PM2.5, meat		621-3805		0.03-0.3		[53]	
Fe_3_O_4_@UiO-66- NH_2_@MON	MSPE	HPLC-FLD	AFT	corn, rice, millet		-		150-870		[54]	
MON-OH-C	SPE	HPLC	FLU	water		-		-		[56]	
H-MON	SPME	GC-MS	SCCPs	water, sediments, organisms		1773-983		3		[57]	
MON-COOH	SPE	HPLC	phenols	water		113-216		130-620		[58]	
B-MON	SPME	GC-MS/MS	bisphenols A, F, S, triclosan	breast milk, infant formula		-		5		[60]	
Fe_3_O_4_@TMON	MSPE	HPLC-FLD	antibiotics	water		256		0.2-0.4		[64]	
Hollow Co@C	MSPE	UV	aromatic pollutants	water		-		-		[65]	
Co@NC-MON-2NH_2_	MSPE	HPLC-UV	PGRs	tomato, mung bean sprout, cucumber		151-196		9-150		[66]	

EFs: enrichment factors. TBBPA: tetrabromobisphenol A; EDCs: endocrine disrupting chemicals; FLU: flumequine; NAD: nadifloxacin; BZDs: benzimidazole fungicides; NSAIDs: non-steroidal anti-inflammatory drugs; BPA: bisphenol A; PCAs: phenoxycarboxylic acids; AFT B1: aflatoxin B1; ST: sterigmatocystin; PUHs: phenylurea herbicides; BTri: 1*H*-benzotriazole; 5-TTri: 5-tolyltriazole; 2,4,6-TCP: 2,4,6-trichlorophenol; OH-PAHs: hydroxylated polycyclic aromatic hydrocarbons; *p*-Npn: *p*-nitrophenol; AFT: aflatoxins; SCCPs: short-chain chlorinated paraffins; PGRs: plant growth regulators.

## 4 总结与展望

MONs因具有比表面积大、稳定性良好、结构可设计等突出优势,在样品前处理领域得到了广泛应用。随着材料合成技术的成熟,室温快速合成法逐渐取代了回流合成法,而且在MONs的功能化修饰上也有更多突破。但还存在一些不足:(1)MONs材料合成中,部分前体和催化剂价格较昂贵,限制了MONs材料的大量合成和应用。选择性能相似、性价比更优的配体试剂,是MONs合成中研究者关注的一个焦点。(2)MONs材料具有刚性共轭平面,对大多数有机物都有萃取效果。如何提高材料的选择性、减少杂质干扰,实现对特定目标物的特异性萃取后续也亟待研究。(3)目前,MONs材料种类还偏少,仍需设计和合成更多具有不同拓扑结构和功能的新型MONs材料。随着对该材料的深入研究,相信MONs及其复合材料在样品前处理及其他领域会拥有巨大的开发潜力和应用前景。
